# The Passing of the Old Guard

**Published:** 1900-09

**Authors:** 


### THE PASSING OF THE OLD GUARD.

YEAR by year, as our Association assembles in the great cities of our land, and the pilgrimages now reaching nearly two-score in the yearly milestones we have passed, the assemblage of the great men who have led veterinary advancement lessens, and the absence of many faces, so familiar to our professional gatherings, grows more and more numerous. When one gazes to-day on the Association group of faces and notes the rapid changes of five, ten, and fifteen years, there are many sad thoughts awakened, and memories dear mingled with regret are wont to rise. Those who yearly prize the photographic group will look in vain for many faces that made in years gone by our place and position sure. The Detroit group has fewer faces from among the old guard than any of its predecessors. Perhaps some of those who yet feel young in membership are now becoming the old guard, and the faces they loved to look upon, the leaders they were proud to follow, the charms of those who fathered this grand Association in 1863, are still to be remembered in the hour of our greater numerical strength, and the value and the peculiar force of the strong fraternal feelings that welded the early membership so firmly should not be forgotten. Its measure of usefulness then had an added wealth in the strong ethical relations they bore to the community at large and fostered among themselves. A newer era in these more modern days has chilled in some measure these strong fraternal relations. The wider circle has dimmed the ethical glass through which we peer, and the spirit of commercialism has not left even our calling without its dangerous imprint.

While these finer lines of ethical commercialism have been drawn, have we not drifted away from strong moorings, that gave safety and refuge from professional dangers, that the strong and rigid rules of to-day are not altogether a barrier of defence.

Let us not forget the old guard in our feeling of greater strength. Let us cherish the charms of their strong fraternal feelings. Let there linger in memory the social bond that made memorable their annual and semi-annual reunions, and while facing the newer and broader problems confronting us to-day, let us cherish the memories that surround Association history as written by the old guard.

